# Prehabilitation: A Catalyst for Transforming Toward Value-Based, Personalized Perioperative Health and Care

**DOI:** 10.3390/jcm14196747

**Published:** 2025-09-24

**Authors:** Brenda H. van Koningsveld-Couperus, Thijs de Rooij, Nico L. van Meeteren, Benedikt Preckel, Markus W. Hollmann, Gertrude J. Nieuwenhuijs-Moeke

**Affiliations:** 1 Department of Anesthesiology, University Medical Center Groningen, University of Groningen, 9713 GZ Groningen, The Netherlands; b.h.van.koningsveld@umcg.nl; 2 Department of Anesthesiology, Amsterdam University Medical Centers, 1105 AZ Amsterdam, The Netherlands; t.derooij@amsterdamumc.nl (T.d.R.); b.preckel@amsterdamumc.nl (B.P.); m.w.hollmann@amsterdamumc.nl (M.W.H.); 3 Top Sector Life Sciences & Health (Health~Holland), 2595 AN The Hague, The Netherlands; meeteren@health-holland.com; 4 Department of Cardiothoragic Surgery, Erasmus Medical Center, 3015 GD Rotterdam, The Netherlands; 5 Netherlands National Committee for the Protection of Animals Used for Scientific Purposes (NCad), 2500 EK The Hague, The Netherlands

**Keywords:** multimodal, value-based care, prehabilitation, postoperative complications, patient empowerment

## Abstract

The growing strain on global healthcare systems, driven by aging populations, rising prevalence of chronic diseases, and workforce shortages, has increased interest in strategies that enhance perioperative outcomes and healthcare sustainability. From this perspective, prehabilitation—a proactive, multimodal approach to enhance patients’ functional, nutritional, and psychological status prior to surgery—has gained attention as a potential contributor to value-based, personalized care. This study aims to synthesize mechanistic rationale, clinical evidence, and system-level considerations for prehabilitation, with particular focus on allostatic capacity and the body’s response to surgical stress. Current evidence shows that prehabilitation may reduce postoperative complications, shorten hospital stays, and improve functional recovery, particularly when interventions are multimodal. However, the existing literature is characterized by methodological heterogeneity and variable quality, seemingly limiting generalizability and large-scale implementation. Further research is required to standardize outcome measures, identify patient subgroups most likely to benefit, and evaluate cost-effectiveness. Integration of prehabilitation into perioperative care pathways will depend on improved mechanistic understanding, robust clinical trials, and alignment with broader health policy and system-level initiatives. Prehabilitation may represent a meaningful step toward value-based and sustainable surgical care, though its implementation must be guided by high-quality evidence and careful consideration of context-specific factors.

## 1. Introduction

The global healthcare system is under increasing strain due to a combination of persistent and emerging challenges, including unequal access to care, rising costs, and a growing shortage of healthcare professionals [1,2]. Many countries face additional pressures from aging populations, a rising burden of chronic diseases, under-resourced infrastructure, and limited access to mental health services [3,4,5]. These factors collectively threaten both the accessibility and quality of care, despite high societal expectations and legal obligations to provide adequate healthcare. In anesthesiology and perioperative care, these systemic challenges manifest as longer waiting times at preoperative outpatient clinics, inefficient operating room utilization, and growing surgical waitlists. The aging population and increasing complexity of patient health further intensify the pressure on both the accessibility and affordability of surgical care [5]. In response, there is growing interest in proactive, preventive strategies aimed at improving patient health and outcomes and alleviating pressure on healthcare systems. Prehabilitation has emerged as a potential solution: a multimodal, proactive approach to optimize patients’ functional, nutritional, and psychological status before surgery. This article explores the role of prehabilitation as an integral component of perioperative health and care. Specifically, it examines how prehabilitation may contribute to measurable health gains for patients and support the sustainability of healthcare delivery.

Although interest in prehabilitation has grown considerably in recent years, the aims, scope, and implementation of this approach remain inconsistently defined. The literature is characterized by methodological heterogeneity, small or narrowly focused studies, and variability in outcome measures, which together limit generalizability and hinder large-scale adoption. Moreover, most reviews focus on single interventions (e.g., exercise or nutrition) rather than considering prehabilitation as a systems-level strategy for value-based, personalized perioperative care. The purpose of this study is, therefore, to synthesize current mechanistic insights, clinical evidence, and policy considerations, and to highlight prehabilitation as a catalyst for transforming perioperative care. By framing prehabilitation in the context of allostatic capacity and the surgical stress response, we aim to define the current knowledge gaps, clarify the rationale for its integration into perioperative pathways, and provide a strategic outlook for future research and implementation.

To support the perspectives presented, a focused literature search was performed in PubMed and The Cochrane Library, targeting studies on prehabilitation and related concepts in the context of preoperative patient optimization and the impact of surgery.

## 2. The Impact of Surgery

**Figure 1:**
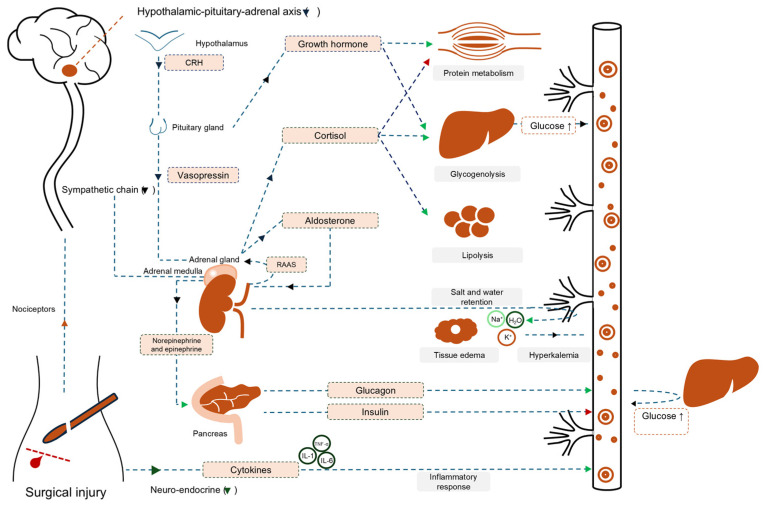
The relationship between surgical tissue damage and the multisystem effects of the surgical stress response. Reproduced with permission from van Beijsterveld C.A.F.M [18]. No modifications were made. Abbreviations: CRH = corticotropin-releasing hormone; H_2_O = water; IL-1 = interleukin-1; IL-6 = interleukin-6; K^+^ = potassium; Na^+^ = sodium; TNF-α = tumor necrosis factor alpha; RAAS = renin–angiotensin–aldosterone system.

Over 310 million surgical procedures are performed worldwide each year [6]. This number is likely to increase with an expanding, ageing population and improved access to healthcare. Annually, an estimated 3 to 6 million patients die within 30 days of surgery [6,7]. Postoperative mortality accounts for 7.7% of all deaths globally, making it the third largest contributor of death after ischemic heart disease and stroke [8]. This mortality results either from underlying diseases, the strain of the operation, or postoperative complications (POCs). Approximately 20% of patients, or >60% in case of high-risk surgery/patients, develop one or more complications in the early postoperative period [9,10]. POCs are associated with increased length of hospital stay and adversely affect disease-free survival, mortality rates, and long-lasting hospital-associated disability. POCs are a burden to patients and society and an important contributor to increasing healthcare costs [11,12]. Several studies on in-hospital costs of complications show average cost increases ranging from $3000 to $130,000 per patient, depending on severity [13]. Even in the absence of POCs, 20–40% of the patients experience loss of physical performance and reduction of quality of life after major surgery [14]. Long-term outcomes from the TRACE study, which included 5473 patients, highlight the magnitude of this issue [15]. One year after surgery, a substantial proportion of patients continued to report functional limitations and impaired well-being. Specifically, 30% were unable to perform daily activities independently, while 40–51% reported persistent pain or discomfort, impaired mobility, or difficulty carrying out routine tasks. These findings underscore that postoperative recovery is frequently more prolonged and complex than is commonly anticipated, with lasting effects on patient independence and quality of life. From a traditional perspective, POCs have mainly been attributed to surgical technique, the nature of the procedure, or the presence of comorbidities. However, the difficulty in adequately identifying patients at increased risk of complications suggests the need for a different perspective, one in which the patient’s response to surgery is considered the primary pathophysiological process. Surgical tissue injury, both direct (e.g., dissection) and indirect (e.g., hypoperfusion, acidosis), triggers a complex biological stress response involving neuroendocrine, metabolic, and immunological changes [16,17] (Figure 1).

**Figure 2:**
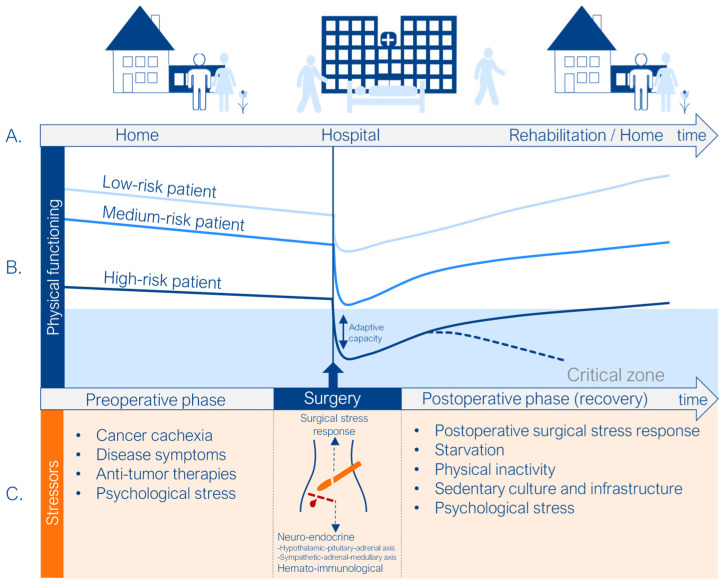
Perioperative stressors and their relationship with physical function along the patient journey. Reproduced with permission from van Beijsterveld C.A.F.M [18]. No modifications were made. (**A**) The patient journey, from home to hospital admission and back home. (**B**) Preoperative physical function is a strong predictor of postoperative recovery. Patients with lower functional status (high-risk patients) are more likely to fall below the critical threshold of adaptive capacity, resulting in higher complication rates and incomplete recovery. (**C**) Physical function is impacted by stressors occurring throughout the pre-, peri-, and postoperative phases, particularly when primary surgery is followed by secondary insults such as complications, reoperations, or readmissions.

Surgical procedures initiate a complex, evolutionarily conserved physiological response aimed at limiting further injury and promoting recovery. This response functions not only to restore homeostasis, but, more accurately, to achieve allostasis, defined as “stability through change”, which enables the organism to adapt to physical, psychological, and social stressors. However, when dysregulated, this adaptive mechanism can become maladaptive, leading to excessive inflammation, organ dysfunction, and immunosuppression, thereby increasing the risk of POCs and contributing to prolonged impairments in physical function [16] (Figure 2). In addition to the surgical insult itself, anesthesia and anesthetic management significantly influence the perioperative stress response and postoperative outcomes. Increasing evidence shows that intraoperative interventions, such as maintenance of normothermia, prevention of acidosis, optimization of fluid therapy, and the use of minimally invasive surgical techniques, as well as the choice of anesthetics, can modulate the stress response and reduce the incidence of POCs [19,20,21,22,23,24]. Anesthetic agents in particular exert immunomodulatory, anti-necrotic, and anti-apoptotic effects [25,26]. Both intravenous anesthetics, such as propofol, and inhalational agents, such as sevoflurane, are routinely used and have been shown to provide protective and anti-inflammatory benefits. Propofol anesthesia has been associated with reduced levels of IL-1, IL-6, and IL-8, as well as decreased oxidative stress [27,28,29,30], whereas sevoflurane has been reported to inhibit the production of IL-1β and IL-6, while attenuating apoptosis-related signaling through proteins such as cytochrome C and caspase-3 [31]. These findings indicate that the anesthetic strategy, alongside surgical and patient-related factors, plays a pivotal role in shaping the biological stress response and subsequent risk of postoperative complications.

The occurrence and consequences of complications are likely driven by a complex interplay of pre-existing comorbidities, physiological reserve (allostatic capacity), genetic and psychosocial factors, and the surgical burden [32,33,34]. A growing body of evidence suggests that preoperative alterations in immune function, including inflammaging (chronic, low-grade inflammation) and immunosenescence (age-related immune decline), contribute significantly to poor outcomes. These states are characterized by impaired immune responsiveness, neuroendocrine and metabolic dysregulation, reduced exercise capacity, and increased frailty. The set point for homeostasis is not fixed, but rather highly individual, shaped over time by both internal factors (e.g., malnutrition, chronic disease, physical deconditioning) and external stressors (e.g., life events, socioeconomic adversity, environmental exposures). The extent to which this set point shifts is referred to as allostatic load, while the capacity to maintain or regain physiological balance is termed allostatic capacity, which typically diminishes with age. When cumulative stress exceeds this adaptive threshold, due to either chronic strain or an acute insult, such as surgery, allostatic overload can occur, leaving the body unable to return to a stable state. Although there is currently no universally accepted and/or standardized method for quantifying allostatic load, it is often assessed using composite indices of cardiovascular, metabolic, inflammatory, and neuroendocrine biomarkers, and when cited, composite indices are illustrative rather than prescriptive [34,35,36]. While associations between high allostatic load and poor postoperative outcomes are recognized, the precise mechanistic pathways remain insufficiently defined. This knowledge gap persists for several key reasons: (1) Most of our current understanding is based on in vitro or animal models, which often lack translational relevance to human physiology. (2) Research has largely focused on individual molecules or pathways, rather than adopting an integrated systems biology perspective. (3) Compared to the postoperative phase, the preoperative period, which may offer crucial opportunities for intervention, has been relatively underexplored.

## 3. Prehabilitation as a Strategy to Enhance Allostatic Capacity

Prehabilitation refers to the proactive optimization of a patient’s functional and physiological status prior to surgery, with the goal of enhancing resilience and minimizing postoperative complications [37]. This approach typically involves multimodal interventions, such as structured exercise programs, nutritional support, psychological preparation, and comorbidity management. Lifestyle modification is an equally important component of prehabilitation. In particular, cessation of smoking and alcohol use should be actively promoted, as both are well-established risk factors for impaired wound healing, cardiopulmonary complications, and delayed recovery [38,39,40]. Evidence shows that integrating structured smoking and alcohol cessation strategies into multimodal prehabilitation programs can reduce postoperative complications and improve recovery trajectories. These lifestyle interventions, therefore, complement exercise, nutrition, and psychological support, collectively aiming to enhance the patient’s capacity to tolerate surgical stress and facilitate a more favorable recovery in collaboration with the patient and their support network. Despite promising conceptual and clinical foundations, the evidence base for prehabilitation remains heterogeneous and, at times, inconclusive, with variability in intervention types, patient populations, outcome measures, and study designs [37]. This heterogeneity has posed challenges for broad implementation, particularly in healthcare systems where cost-effectiveness, scalability, and reproducibility are essential for policy integration. However, when heterogeneous studies, differing in methods and contexts, demonstrate consistent benefits, this heterogeneity can be interpreted as a strength, indicating robustness and generalizability of the evidence base. Nonetheless, there is growing consensus among international experts on the need for a standardized, evidence-based framework for prehabilitation and perioperative optimization strategies.

A key target of prehabilitation is the improvement of aerobic fitness, which reflects the integrated capacity of the respiratory, cardiovascular, and muscular systems to deliver and utilize oxygen. Aerobic capacity is commonly assessed using cardiopulmonary exercise testing (CPET), which provides objective parameters such as peak oxygen uptake (VO_2_ peak) and the ventilatory anaerobic threshold (VAT). A VAT below 11 mL/kg/min is associated with an increased risk of postoperative complications following major abdominal surgery [41,42,43]. Although CPET is considered the gold standard for evaluating aerobic fitness, it is resource-intensive and not universally accessible. Alternative tests, such as the (modified) steep ramp test (SRT) or the incremental shuttle walk test (ISWT), offer more practical and cost-effective methods for assessing aerobic capacity in the clinical setting [44]. The SRT, in particular, has shown strong correlation with VO_2_ peak values from CPET and is significantly shorter in duration, making it a reliable and time-efficient surrogate [45]. However, standardized threshold values for risk stratification based on SRT performance have yet to be firmly established. In addition to aerobic fitness, other domains, such as muscle strength, nutritional status, and psychosocial resilience, are increasingly recognized as important contributors to surgical outcomes. The impact of prehabilitation on these domains varies across studies and appears highly dependent on patient characteristics, intervention components, and timing. While many studies have reported reductions in complications, shorter hospital stays, and improved functional recovery, the effects remain inconsistent, underscoring the need for further research using standardized methodologies.

Beyond its role in improving aerobic capacity and functional status, prehabilitation may also serve as a biologically targeted intervention to modulate the stress response to surgery at the molecular and cellular level [46]. Surgical trauma initiates a complex inflammatory cascade, which, when dysregulated, contributes to the development of postoperative complications such as infection, sepsis, and organ dysfunction. Emerging evidence suggests that multimodal prehabilitation, including exercise and nutritional interventions, can positively influence immune-metabolic pathways that regulate this response. Specifically, prehabilitation has been shown to impact two key biological mechanisms: the regulation of hypoxia-inducible factors and the metabolism of ceramides, a class of bioactive sphingolipids associated with inflammation and metabolic dysfunction [46,47,48]. Exercise-induced remodeling of skeletal muscle promotes anti-inflammatory signaling and improves immune surveillance by modulating these pathways, while nutritional support can further enhance metabolic flexibility and reduce systemic inflammation [49,50]. This molecular remodeling may help mitigate the effects of inflammaging and immunosenescence, particularly in older or frail patients, thereby expanding their allostatic capacity [51]. Additionally, prehabilitation appears to influence key immune cell populations, such as regulatory T cells and natural killer cells, which are essential for modulating inflammation and preventing tumor progression [52]. Despite these promising insights, many of the underlying mechanisms remain to be elucidated, and further translational research is needed to clarify how immune-metabolic changes induced by prehabilitation translate into improved clinical outcomes.

## 4. Scientific Justification for Prehabilitation

Before widespread implementation of prehabilitation programs, it is critical to evaluate their effectiveness, clinical relevance, and cost-efficiency. Given that prehabilitation is resource-intensive, determining which patients benefit most and which program components are essential is key to ensuring value-based care. Although the concept of prehabilitation in healthcare has been recognized since the 1990s, its scientific foundation remains limited, with many studies relying on observational designs and even randomized controlled trials (RCTs) reporting inconsistent results. The heterogeneity of study populations, interventions, and outcome measures significantly contributes to these discrepancies. In response, an international consortium of prehabilitation experts has called for a standardized research and clinical framework that supports personalization of care, while ensuring consistency in screening, assessment, and implementation. This includes the use of validated instruments and harmonized outcome measures to assess effectiveness across settings [53].

One illustrative example is the PREHAB RCT conducted by Molenaar et al., which evaluated a four-week multimodal prehabilitation program for patients undergoing colorectal surgery [54]. The intervention included high-intensity interval training (three times per week), home-based exercises, nutritional counseling, psychological support, and smoking cessation guidance. Among 251 patients, those who completed the prehabilitation program experienced fewer severe postoperative complications and demonstrated faster recovery compared to standard care controls.

A systematic review and meta-analysis by Perry et al. synthesized data from 178 studies on prehabilitation in major surgery [55]. It found that risk factors for postoperative complications were largely consistent across surgical domains, and that four types of interventions, being respiratory muscle training, physical exercise, immunonutrition, and multimodal programs, yielded favorable outcomes. Notably, infectious complications were reduced by 37% with immunonutrition, and pulmonary complications decreased by 45% with respiratory training and 46% with physical training. Additionally, hospital stays were shortened by an average of 1.5 to 2 days. However, the overall quality of evidence was rated as low to very low, due to small sample sizes, intervention heterogeneity, and possible expectation bias.

Similarly, Steinmetz et al. conducted a meta-analysis on prehabilitation in cardiac surgery, including six studies with 665 patients [56]. Interventions consisted of ≥90 min/week of physical training, often combined with psychosocial or lifestyle support, over a minimum of two weeks. The results showed improvements in the 6-min walk test (6MWT), a one-day reduction in hospital stay, and a lower incidence of postoperative atrial fibrillation in patients under 65. A trend toward fewer pulmonary complications was also noted. However, results were constrained by risk of bias and substantial variation in study design and populations.

In the domain of hepato-pancreato-biliary surgery, a meta-analysis of four studies (419 patients) suggested benefits of prehabilitation in terms of reduced hospital stays and readmission rates [57]. Yet again, significant heterogeneity in patient selection and intervention design highlighted the need for more rigorous investigation.

A recent Cochrane review by Molenaar et al. assessed three RCTs involving 250 patients and concluded that while prehabilitation may improve preoperative functional capacity, its effects on complications, readmissions, and quality of life were small or uncertain [58]. These limitations, especially small sample sizes, intervention variability, and high risk of bias, underscore the need for cautious interpretation and further study.

This conclusion is reinforced by an umbrella review from McIsaac et al., which included 55 systematic reviews covering approximately 28,363 patients [59]. The authors found low to very low quality of evidence supporting benefits of prehabilitation, particularly in cardiovascular and abdominal surgeries, and no consistent benefits in orthopedic or oncologic procedures.

Most recently, a 2025 network meta-analysis of 186 RCTs involving 15,684 patients evaluated a broad range of prehabilitation strategies across surgical specialties. Isolated interventions in physical training, nutrition, or their combinations (including psychosocial support) were associated with a reduction in postoperative complications, particularly physical training: OR 0.50, nutrition: OR 0.62, and combined interventions: OR 0.64. For hospital length of stay, the greatest reduction was observed with physical training plus psychosocial support (−2.44 days), followed by physical training plus nutrition (−1.22 days). Only the latter was supported by moderate-certainty evidence. Combined approaches also showed the most improvement in health-related quality of life (+3.48 points) and functional recovery, as measured by an increase of 43 m in the 6MWT [60]. A key limitation of this network meta-analysis is its limited assessment of the quality and contextual delivery of prehabilitation interventions. Factors such as supervision, adherence, individualization, and behavioral strategies, which critically influence their effectiveness, were not sufficiently explored. This highlights the need to evaluate not just methodological rigor, but also the fidelity and depth of intervention.

To illustrate the current state of knowledge, a summary of available literature is shown in Table 1.

## 5. Reflection and Future Perspectives

The evidence base for prehabilitation is steadily expanding, but it remains insufficiently consistent to justify uniform routine implementation across healthcare systems. A major barrier lies in the heterogeneity of study designs, populations, interventions, and outcomes. While often seen as a weakness, this variation can also be a strength: when diverse trials nevertheless converge on benefits such as reduced pulmonary complications and improved functional capacity, the evidence gains robustness and generalizability. By contrast, null findings are typically seen in low-risk cohorts, after short interventions, or in studies with poor adherence. The question, therefore, is not *whether* prehabilitation works, but *what works for whom, and under which conditions*.

Future studies should prioritize stratified analyses (frailty, baseline fitness, inflammageing profiles), systematically report intervention fidelity (dose, supervision, adherence), and adopt core outcome sets that span both short- and long-term domains, including complications, function, participation, quality of life, and equity.

Prehabilitation itself covers a broad spectrum—from isolated physical training to multimodal programs incorporating nutrition, sleep optimization, cognitive training, smoking and alcohol cessation, and psychological counselling. This diversity complicates consensus on what constitutes an optimal program, yet international momentum for integrating prehabilitation into perioperative care continues to build. In the Netherlands, for example, the Dutch Society of Surgery (NVvH) and the Dutch Society of Anesthesiology (NVA) have explicitly included prehabilitation in their strategic agendas, signaling recognition of its future role in surgical care [61,62].

The ongoing debate around effectiveness highlights the urgent need for more high-quality trials, cost-effectiveness studies, and mechanistic insights into biological, behavioral, and clinical variability. Without these, healthcare providers lack the tools to stratify risk and personalize interventions. This gap is also emphasized in Dutch national policy documents such as the Integral Care Agreement (Integraal Zorgakkoord, IZA) and programs like Fit4Surgery, which aim to promote prevention and value-based care. An international Delphi study further reflects this consensus, identifying key research priorities in prehabilitation. These include identifying patient subgroups most likely to benefit, establishing standardized outcome measures, and developing biomarkers or predictive models to guide clinical decision-making [63] (Table 2).

The call for precision in prehabilitation is not merely academic, it is a prerequisite for achieving scalable, sustainable, and equitable implementation

From a health-economic perspective, prehabilitation shows promise. A Dutch budget impact analysis estimated that a national multimodal program for colorectal surgery could save €12.8 million annually, or €64.3 million over five years [64]. These gains are partly dependent on adherence and delivery models, but could grow with greater personalization, outcome monitoring, and integration into routine pathways. Still, not all patients will benefit equally; identifying determinants of success and failure—clinical, behavioral, and social—remains essential.

Globally, health systems face rising demand, ageing populations, workforce shortages, and widening disparities. In this context, prehabilitation aligns closely with movements toward proactive, preventive, and person-centered care, echoing frameworks such as value-based healthcare, the WHO’s surgical strategies, and the Dutch IZA’s national focus on prevention and efficiency [65]. Yet, it is not consistently recognized as “state-of-the-art” science or practice. In the Netherlands, for example, multimodal prehabilitation for colorectal cancer patients is not yet reimbursed as a bundled package, despite most components already being covered individually. The Dutch National Health Care Institute has, therefore, called for large-scale, pragmatic RCTs in high-risk patients using outcomes meaningful to both patients and clinicians [66]. Such trials should adopt a systems approach spanning the perioperative period, combining multidimensional data with real-world evidence, artificial intelligence, and wearable technologies.

### 5.1. Value-Based and Personalized Care

In a value-based paradigm, the true currency is patient-relevant outcomes, function, participation, and quality of life, measured across both short and long horizons. Personalization requires tailoring program intensity, modality, and delivery models (supervised, hybrid, remote) to individual risk and context, including health literacy, social support, geography, and digital access. Importantly, prehabilitation must be embedded in shared decision-making, including consideration of non-surgical alternatives for high-risk individuals.

### 5.2. Stakeholder Perspectives

*Patients and families*: Prehabilitation reframes patients as active partners, improving preparedness, reducing anxiety, and fostering agency. Key enablers include culturally appropriate materials, flexible delivery models, and caregiver involvement.

*Clinicians and teams:* Success depends on multidisciplinary integration, early identification, clear referral pathways, and feedback loops linking preoperative progress with intra- and postoperative management. Professional development must include behavior change, communication, and data literacy.

*Industry and innovation partners:* eHealth platforms, wearables, and MedTech innovations can support precision and scalability. Public–private partnerships should adhere to value-based principles with transparent outcome reporting and safeguards for equity and privacy.

### 5.3. Innovation Horizons

*AI-driven risk stratification:* Predictive models combining clinical, functional, and biomarker data must be externally validated and bias-audited.

*Wearable monitoring:* Metrics such as activity, heart rate variability, and sleep can provide real-time insights and trigger early interventions.

*Real-world data ecosystems:* Interoperable, privacy-preserving platforms can enable continuous learning and model development across centers.

*Equity by design:* Digital solutions must be inclusive, multilingual, offline-compatible, and sensitive to social determinants of health.

### 5.4. Measuring What Matters

Traditional outcomes (complications, readmissions, length of stay) are necessary but insufficient. Value-based evaluation requires measurement of:

Functional ability (WHO framework);

PROMs and patient-reported experience measures (PREMs) across multiple time points;

Durability of lifestyle change;

Equity of access and outcomes;

Cost-effectiveness and cross-sector impact.

A core outcome set, co-created with patients and professionals, should be mandated for both trials and real-world programs.

### 5.5. A Roadmap for Scaling Prehabilitation

*Policy and reimbursement:* Establish bundled payments or add-on tariffs, with safeguards for equitable access.

*Standards and guidelines:* Define minimum program elements, referral triggers, and timing; publish quality indicators.

*Service models:* Provide tiered delivery (center-based for highest risk, hybrid for moderate, digital for lower risk) integrated with enhanced recovery after surgery protocols.

*Workforce and training:* Build multidisciplinary capacity with expertise in behavior change, data, and implementation science.

*Data and governance:* Develop interoperable standards, consent models, privacy safeguards, and transparent dashboards, within a learning health-system framework.

### 5.6. Cost and Resource Implications

Beyond scientific and clinical uncertainties, the cost and resource implications of prehabilitation must also be acknowledged. Multimodal programs require structured exercise supervision, nutritional and psychological support, and close coordination between specialties, all of which may increase short-term healthcare expenditures. Although several budget impact analyses suggest that reductions in postoperative complications, shorter hospital stays, and improved recovery could generate substantial long-term savings, robust cost-effectiveness data are still scarce. Moreover, implementation may not be equally feasible across healthcare systems, and disparities in patient access, adherence, and local infrastructure could limit scalability. Addressing these challenges will require pragmatic health-economic evaluations, prioritization of high-risk populations most likely to benefit, and integration of prehabilitation into existing perioperative care pathways to optimize resource use.

## 6. Limitations

While this perspective provides a synthesis of mechanistic rationale, clinical evidence, and system-level considerations, several limitations should be acknowledged. First, the evidence base for prehabilitation remains heterogeneous, with variability in study design, intervention components, patient populations, and outcome measures. This limits the ability to draw firm conclusions or establish standardized protocols. Second, most available studies are underpowered, short-term, or conducted in selected populations, which restricts generalizability. Third, the mechanistic insights into how prehabilitation modulates the surgical stress response, including immune and metabolic pathways, are still incomplete and largely derived from small-scale or preclinical studies. Finally, as a perspective article, our synthesis is inherently selective and interpretative rather than a systematic review; while we aimed for breadth and balance, important nuances from individual trials may not be fully captured.

## 7. Conclusions: A Strategic Path Forward

Prehabilitation should be recognized not as a stand-alone intervention, but as a cornerstone of adaptive, value-based perioperative care. Its potential lies in fostering long-term resilience and patient empowerment, physically, mentally, and socially. Achieving this vision demands rigorous trials, real-world data integration, and mission-driven collaboration among patients, clinicians, policymakers, insurers, and industry. Ultimately, the critical question remains: *Which patient needs which form of prehabilitation, and how can benefits be made both immediate and lasting?*

Only by closing evidence gaps, aligning incentives, and embedding prehabilitation within a learning healthcare system can it fulfil its promise as a global standard of high-value, personalized, and sustainable surgical care.

## Figures and Tables

**Table 1:** Summary of literature.

Year	First Author	Study Design	Study Population	Prehabilitation Method	Results (Statistically Significant Only)
2021	Dagorno [57]	Systematic review of 3 RCTs and 1 propensity score matched cohort	Adult patients undergoing HPB surgery	1.5 to 4 weeks preoperative physical exercise (4 studies) Nutritional support (3 studies)	Length of hospital stay (MD (95% CI)) Prehabilitation −1.19 (−1.56, −0.81 fixed effect model) and −4.37 (−8.86, 0.13) random effect model)
2021	Perry [55]	Systematic review of 178 RCTs	Adult patients undergoing major surgery and preoperative intervention	(Immuno)nutritional support (51 studies) IMT/IS (30 studies) Physical exercise (27 studies) Multimodal (25 studies)	Length of hospital stay (MD (95% CI)) Immunonutritional support −2.11 (−3.07, −1.15) IMT −1.81 (−2.31, −1.31) Multimodal −1.67 (−2.31, −1.03) Postoperative pulmonary complications (RR (95% CI)) Physical exercise 0.54 (0.39–0.75) IMT 0.55 (0.38–0.80)
2022	Molenaar [58]	Systematic review of 3 RCTs	Adult patients undergoing colorectal surgery	At least two preoperative interventions (physical exercise combined with any nutritional support or mental support or substance use or smoking cessation	6MWT preoperatively (MD (95% CI)) Prehabilitation 24.91 (CI 11.24, 38.57)
2022	McIsaac [59]	Umbrella review of systematic reviews	Adult patients undergoing surgery	Physical exercise (14 SRs), (Immuno)nutritional support (7 SRs), Psychosocial support (3 SRs), Multimodal programs also included	Postoperative complications (OR (95% CI) Physical exercise 0.50 (0.39–0.64) Nutritional support 0.62 (0.50, 0.77) Physical exercise, nutrition, and psychosocial support 0.64 (0.45, 0.92) Length of hospital stay (MD (95% CI)) Physical exercise and psychosocial support −2.44 (−3.85, −1.04) Nutritional support −0.99 (−1.49, −0.48) Physical exercise −0.93 (−1.27, −0.58) Quality of life (MD (95% CI)) Physical exercise, nutrition, and psychosocial support 3.48 (0.82, 6.14) Physical exercise 2.29 (0.96, 3.62)
2023	Steinmetz [56]	Systematic review of 6 studies	Adult patients undergoing cardiac surgery (CABG and/or valve surgery)	2–8 weeks preoperative physical exercise (6 studies) Multimodal (5 studies)	6MWT preoperatively (MD (95% CI)) Prehabilitation 75.36 (95% CI 13.65, 137.07) 6MWT postoperatively (MD (95% CI)) Prehabilitation 30.54 (8.45, 52.63) Atrial fibrillation < 65 years of age (RR (95% CI)) Prehabilitation 0.34 (0.14, 0.83)
2023	Molenaar [54]	RCT (PREHAB trial)	Adult patients undergoing colorectal cancer surgery	Multimodal (exercise, nutrition, psychological support) 251 patients (prehabilitation n 123, standard of care n 128)	Severe complications (CCI > 20): 17.1% vs 29.7%, OR 0.47 (95% CI 0.26–0.87) Medical complications: 15.4% vs 27.3%, OR 0.48 (95% CI 0.26–0.89 ICU admission: 3.3% vs 10.9%, OR 0.29 (95% CI 0.09–0.89)
2025	McIsaac [60]	Systematic review of 186 RCTs	Adult patients undergoing surgery	Physical exercise (133 studies) (Immuno)nutritional support (68 studies) Psychosocial support (31 studies)	Postoperative complications (OR (95% CI)) Physical exercise 0.50 (0.39–0.64) Nutritional support 0.62 (0.50, 0.77) Physical exercise, nutrition, and psychosocial support 0.64 (0.45, 0.92) Length of hospital stay (MD (95% CI)) Physical exercise and psychosocial support −2.44 (−3.85, −1.04) Nutritional support −0.99 (−1.49, −0.48) Physical exercise −0.93 (−1.27, −0.58) Quality of life (MD (95% CI)) Physical exercise, nutrition, and psychosocial support (3.48 (0.82, 6.14)) Physical exercise 2.29 (0.96, 3.62)

Abbreviations: CI = confidence interval, HPB = hepato-pancreatic-biliary surgery, IMT = inspiratory muscle training, IS = incentive spirometry, MD = mean difference, OR = odds ratio, RCT = randomized controlled trial, RR = relative risk, SR = systematic review.

**Table 2:** Top 10 research priorities in prehabilitation according to international Delphi consensus [63].

Research Priority	Description
1. Effectiveness of prehabilitation on surgical outcomes	Investigate how prehabilitation affects postoperative complications, 30-day mortality, length of hospital stay, intensive care unit admission, and readmissions.
2. Identifying patient groups who benefit most	Determine which patients (based on factors like age, sex, fitness, frailty) benefit most from prehabilitation.
3. Optimal composition of prehabilitation programs	Understand the added value of a multimodal approach (e.g., nutrition, physical training, psychosocial support, medical optimization).
4. Standardization of outcome measures	Develop a core set of outcomes to uniformly assess and compare prehabilitation effectiveness in both practice and research.
5. Effect on functional outcomes	Study how prehabilitation impacts physical performance, mobility, and daily living after surgery.
6. Impact on patient-reported outcomes	Examine how prehabilitation contributes to quality of life, well-being, and other patient-reported outcome measures (PROMs).
7. Cost-effectiveness of prehabilitation	Conduct cost–benefit analyses of interventions and determine the resources required to implement prehabilitation programs.
8. Improving adherence and engagement	Develop strategies to enhance patient participation and persistence in prehabilitation programs.
9. Applicability during neoadjuvant therapies	Investigate whether prehabilitation can be safely and effectively implemented during chemotherapy or other pre-treatment regimens.
10. Delivery models and accessibility	Compare delivery models such as telehealth, home-based programs, center-based, and community models—particularly in rural vs. urban settings.

## Abbreviations

6MWT	6 min walk test
CPET	Cardiopulmonary exercise testing
ISWT	Incremental shuttle walk test
IZA	Dutch Integral Care Agreement
OR	Odds ratio
POCs	Postoperative complications
PREMs	Patient-reported experience measures
PROMs	Patient-reported outcome measures
RCT	Randomized controlled trial
SRT	Steep ramp test
VAT	Ventilatory anaerobic threshold
WHO	World Health Organization
